# Vaccination with an Attenuated Ferritin Mutant Protects Mice against Virulent *Mycobacterium tuberculosis*

**DOI:** 10.1155/2015/385402

**Published:** 2015-08-03

**Authors:** Selvakumar Subbian, Ruchi Pandey, Patricia Soteropoulos, G. Marcela Rodriguez

**Affiliations:** ^1^Laboratory of Mycobacterial Immunity and Pathogenesis, Rutgers, The State University of New Jersey, 225 Warren Street, Newark, NJ 07103, USA; ^2^Public Health Research Institute at Rutgers Biomedical and Health Sciences, Rutgers, The State University of New Jersey, 225 Warren Street, Newark, NJ 07103, USA

## Abstract

*Mycobacterium tuberculosis* the causative agent of tuberculosis affects millions of people worldwide. New tools for treatment and prevention of tuberculosis are urgently needed. We previously showed that a ferritin (*bfrB*) mutant of *M. tuberculosis* has altered iron homeostasis and increased sensitivity to antibiotics and to microbicidal effectors produced by activated macrophages. Most importantly, *M. tuberculosis* lacking BfrB is strongly attenuated in mice, especially, during the chronic phase of infection. In this study, we examined whether immunization with a *bfrB* mutant could confer protection against subsequent infection with virulent *M. tuberculosis* in a mouse model. The results show that the protection elicited by immunization with the *bfrB* mutant is comparable to BCG vaccination with respect to reduction of bacterial burden. However, significant distinctions in the disease pathology and host genome-wide lung transcriptome suggest improved containment of Mtb infection in animals vaccinated with the *bfrB* mutant, compared to BCG. We found that downmodulation of inflammatory response and enhanced fibrosis, compared to BCG vaccination, is associated with the protective response elicited by the *bfrB* mutant.

## 1. Introduction

Tuberculosis (Tb) continues to be one of the most deadly infectious diseases, causing more than one million deaths in the world per year. The variable efficacy of the BCG vaccine, the synergy between HIV and* M. tuberculosis* (Mtb), and the increase in drug-resistant Mtb strains underscore the urgent need for effective vaccines against Tb.

Iron plays a critical role in the pathogenesis of many organisms including Mtb. It is the preferred redox cofactor in several basic cellular processes, but due to its insolubility and potential toxicity under physiological conditions, iron is a limiting nutrient in the host environment. Therefore, successful pathogens must possess high affinity iron acquisition systems and exert strict control over intracellular free iron to establish a productive infection. In addition to its ability to induce iron acquisition systems, iron limitation encountered in the host is a signal for pathogen's induction of virulence determinants and reprogramming of many cellular processes including central metabolism, secretion, surface remodeling, stress responses, and membrane vesicle generation [1, 2]. Thus, due to its impact on the pathogen's physiology, iron homeostasis can influence many host-pathogen interactions.

Previously, we demonstrated that Mtb requires the iron storage protein ferritin (BfrB), for its adaptation to changes in iron availability [3]. When available, iron is safely stored in ferritin and released as needed to overcome iron limitation. Lack of storage results in free iron mediated toxicity and increased vulnerability to oxidative and nitrosative stress. In mice lungs, Mtb lacking* bfrB* (Δ*bfrB*) proliferates initially, in the first 4 weeks, but it succumbs to the effects of the adaptive immune response failing to establish a chronic infection [3]. Additionally, though Δ*bfrB* disseminates to the spleen, it fails to colonize the liver [3]. Thus, although unable to establish a successful infection Δ*bfrB* persists long enough to stimulate host immunity suggesting that this strain could serve as a potential vaccine candidate.

In this study, we tested whether subcutaneous vaccination with Δ*bfrB* could protect mice against a subsequent aerosol challenge with virulent Mtb. The results show that immunization with the Δ*bfrB* stimulates protective immunity associated with reduced disease pathology and better containment of the infection compared to vaccination with BCG. Genome-wide transcriptome analysis showed a distinct expression pattern of significantly differentially expressed genes (SDEG) between the Δ*bfrB* and BCG-vaccinated, Mtb-infected mice lungs. Our network/pathway analysis of SDEG revealed significant downregulation of inflammatory response and activation of fibrosis network genes in the Δ*bfrB*, compared to BCG vaccinated, Mtb-infected mice lungs, which is potentially associated with the improved protection by the former vaccine strain. The results provide a framework for the identification of new immunological correlates and mechanisms of protection, relevant for the design of better Tb prevention strategies.

## 2. Materials and Methods

### 2.1. Animal Ethics Statement

All animal procedures mentioned in this study were approved by the Rutgers Institutional Animal Care and Use Committee (IACUC) and all possible steps were taken to avoid animal suffering at each stage of the experiments.

### 2.2. Bacterial Strains and Chemicals

The wild type and Δ*bfrB* strains of Mtb H37Rv were grown in Middlebrook 7H9 media (Difco BD, Sparks, MD) as described previously [3]. Stock cultures were prepared at mid-log growth phase and stored at −80°C until they were ready to use. All chemicals were purchased from Sigma (Sigma-Aldrich, St. Louis, MO), unless specified otherwise. BCG-Pasteur strain was obtained from ATCC.

### 2.3. Mouse Vaccination and Infection

Female C57BL/6 mice (*n* = 15) were vaccinated subcutaneously with 1 × 10^6^ CFU of Δ*bfrB* or BCG suspended in 0.1 mL PBS containing 0.05% Tween 80 (PBST). A control group of mice was mock-vaccinated with PBST. At 4 or 8 weeks postvaccination, groups of 5 mice (*n* = 5) were aerosol infected with Mtb H37Rv as described earlier [3]. Briefly, Mtb aerosols were generated by a Lovelace nebulizer (In-tox Products, Albuquerque, NM) with a 10 mL bacterial suspension of about 1 × 10^6^ bacilli/mL in saline containing 0.04% Tween 80 and the mice were exposed to the aerosol for 30 minutes which results in approximately 100 colonizing CFUs per lung. A separate group of vaccinated but uninfected mice were included as controls. Infected mice were sacrificed 4 weeks postinfection and lungs and spleen were removed. Portions of the tissue were homogenized in PBST and serial dilutions of the homogenates were plated onto Middlebrook 7H10 agar (Difco BD, Sparks, MD) to determine the number of bacterial CFUs. Portions of mice lungs were also stored immediately at −80°C for total lung RNA isolation or fixed in 10% formalin for histological analysis.

### 2.4. Histopathology

The formalin-fixed lung portions from vaccinated and vaccinated-Mtb-infected mice (*n* = 5 per group) were paraffin embedded, cut into 5 *μ*M slices, and used for histopathological analysis. Lung sections were stained either with haematoxylin and eosin (H&E), Mason's trichrome, or Ziehl-Neelsen staining method for assessing the pathology, tissue remodeling and fibrosis, and the presence of acid-fast bacilli (AFB), respectively. Stained lung sections were analyzed and photographed in a Nikon Microphot-FX microscope (Nikon Instruments, Melville, NY). Morphometric analysis was performed on the H&E stained histologic sections to determine the area of granulomas in the lungs of Mtb-infected mice using Sigmascan Pro software, following the instructions supplied by the manufacturer (Systat Software, Inc., San Jose, CA).

### 2.5. Total RNA Isolation

Total lung RNA from vaccinated and vaccinated plus Mtb-infected mice (*n* = 5 per group) was extracted and purified to remove contaminating DNA, using the RNeasy Midi kit (Qiagen Inc., Valencia, CA) following the manufacturer instructions. The quality and quantity of purified RNA was estimated using a Nanodrop instrument (Nanodrop, Wilmington, DE).

### 2.6. Microarray Gene Expression Procedure and Data Analysis

Total RNA extracted from each of the BCG or Δ*bfrB* vaccinated and Mtb-infected mouse lungs (*n* = 3 per group) was processed separately for microarray analysis as reported earlier [4]. Gene expression profiling was performed using the Mouse Gene 2.0 ST Array slides and the GeneChip whole transcript (WT) protocol following the manufacturer's protocol (Affymetrix, Santa Clara, CA). Briefly, total RNA (250 ng) was used for cDNA synthesis. The cDNA was fragmented and end-labeled with biotin. The biotin labeled cDNA was hybridized to the array for 16 hours at 45°C using the GeneChip Hybridization Oven 640. Washing and staining with streptavidin-phycoerythrin was performed using the GeneChip Fluidics Station 450. Images were acquired using the Affymetrix Scanner 3000 7G Plus and the Affymetrix Command Console Software (Affymetrix, Santa Clara, CA). The arrays were normalized using robust multiarray (RMA) method. Gene expression data from three independent samples per group was cumulated, averaged, annotated, and processed at group level (i.e., BCG vaccinated and Mtb-infected versus Δ*bfrB* vaccinated and Mtb-infected) using Partek Genomics Suite (Partek Inc., St. Louis, MO). We applied one-way ANOVA with equal variance between comparator groups. Significantly differentially expressed genes (SDEG) were derived using 5% false discovery rate (FDR). The list of SDEG was analyzed for gene ontology and pathway/network derivation using ingenuity pathway analysis (IPA; Ingenuity Systems, Redwood City, CA). A *z*-score based gene ontology algorithm was used from IPA to categorize the biological functions affected by SDEG. This algorithm identifies biological functions expected to be increased (activated) or decreased (suppressed), in the user datasets, based on the nonrandom expression pattern of genes in the dataset, relative to the knowledgebase from published results. Thus, a *z*-score of ≥2 indicates activation of a biological function, while a *z*-score of ≤2 shows suppression of a biological function. The microarray gene expression data was submitted to Gene Expression Omnibus (GEO).

### 2.7. Quantitative Real-Time PCR (qPCR)

The mouse lung total RNA utilized for microarrays was also used for qPCR experiments. For reverse transcription, a ThermoScript RT-PCR system with random hexamer primers was used according to the manufacturer instructions (Life Technologies, Grand Island, NY). Briefly, 200 ng of total lung RNA from each mouse was used for cDNA synthesis. Control reactions without reverse transcriptase were also performed to exclude significant DNA contamination. The amplified cDNA samples were diluted 50-fold and used for qPCR with a SYBR green qPCR super Mix Universal (Life Technologies, Grand Island, NY) in a Stratagene Mx3005p system (Agilent Technologies, Santa Clara, CA). The 10 *μ*L PCR mixture contained 1.5 *μ*L (5 ng) of cDNA template, 5 *μ*L of SYBR Green Master Mix, 2 *μ*L of primers (10 pM), and 1.5 *μ*L nuclease-free water. The PCR conditions were one cycle of 10 min at 94°C followed by 40 cycles of amplification, each with 95°C for 30 s, 55°C for 30 s, and 72°C for 30 s. The oligonucleotide primers used for qPCR are listed in Supplementary Table  1 in Supplementary Material available online at http://dx.doi.org/10.1155/2015/385402. Threshold cycle (Ct) values of test and control (*Gapdh*) genes were measured and used to calculate the fold change in expression using Mx4000 software (Agilent Technologies, Santa Clara, CA).

## 3. Results

### 3.1. Growth of Mtb in Δ*bfrB* or BCG Vaccinated Mice

**Figure 1:**
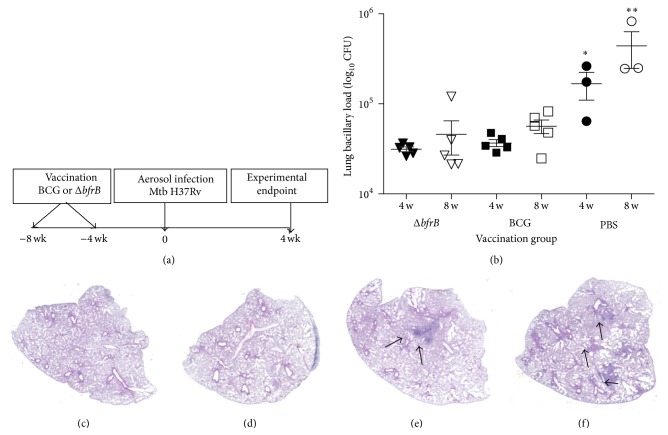
Experimental design, lung bacillary burden, and gross pathology of vaccinated and uninfected or Mtb-infected mice. (a) Schema of the mice vaccination schedule and Mtb infection experiment. (b) Lung bacillary load in mice infected with virulent Mtb H37Rv after vaccination with PBS (sham) or BCG or Δ*bfrB* for 4 (^*∗*^ *P* = 0.033) or 8 weeks (^*∗∗*^ *P* = 0.035). Gross lung pathology of vaccinated and uninfected or Mtb-infected-mice. (c) H&E stained lung section of BCG vaccinated (for 8 weeks) and uninfected mice. (d) H&E stained lung section of Δ*bfrB* vaccinated (for 8 weeks) and uninfected mice. (e) H&E stained lung section of BCG vaccinated (for 8 weeks) and Mtb-infected (for 4 weeks) mice. The arrows in (e) show a multifocal, coalescent granuloma. (f) H&E stained lung section of Δ*bfrB* vaccinated (for 8 weeks) and Mtb-infected (for 4 weeks) mice. The arrows in (f) show multiple, small granulomas.

We have shown previously that, after aerosol exposure, Δ*bfrB* replicates in the mice lungs during the first 4 weeks of infection but is unable to persist and establish a chronic infection. We postulated that the inability of Δ*bfrB* to persist in the infected mice lungs was due to the effects of the adaptive immune response and hypothesized that the transient replication of Δ*bfrB* might stimulate an immune response that could protect against subsequent infection with a fully virulent Mtb. To test this, groups of mice (*n* = 5) were vaccinated with a single dose of Δ*bfrB* or the standard BCG vaccine and at four or eight weeks postvaccination a group of mice were sacrificed to determine CFUs of each vaccine strain in the lungs whereas a second group of mice were challenged via aerosol infection with virulent wild type H37Rv (Figure 1(a)). Enumeration of lung CFUs from vaccinated, uninfected mice lungs show that at the high dose used for subcutaneous administration the attenuation of Δ*bfrB* and BCG was comparable (Supplementary Figure  1). Among the Mtb-infected mice, those vaccinated with Δ*bfrB* or BCG had significantly less number of bacilli in their lungs and spleen, compared to the sham (PBS-)vaccinated controls (Figure 1(b) and Supplementary Figure  2). A further reduction in bacillary load was observed in Δ*bfrB* compared to the BCG in the 8 weeks vaccinated, Mtb-infected mice lungs, though this difference was not statistically significant (*P* = 0.63). Thus, we conclude that Δ*bfrB* vaccinated mice controlled bacterial multiplication in the lungs to a similar extent as the BGC vaccinated mice. Since there was no significant difference in the level of protection between animals vaccinated for 4 or 8 weeks prior to challenge, we decided to focus on the longer lasting response of animals vaccinated for 8 weeks before infection, for subsequent analyses.

### 3.2. Histopathology of Δ*bfrB* or BCG Vaccinated Mtb-Infected Mice Lungs

To examine the gross pathology of the Δ*bfrB* or BCG vaccinated and Mtb-infected mice, we examined the H&E stained lung sections from the respective animals (Figures 1(c)–1(f)). No obvious granulomas were found in the Δ*bfrB* or BCG vaccinated, uninfected mice lungs, while well-formed granulomas were noted in the vaccinated-Mtb-infected mice lungs. The lung granulomas appeared bigger and more cellular in the BCG vaccinated, compared to the Δ*bfrB* vaccinated mice, where multiple, smaller granulomas were found. Thus, suggesting that vaccination with these two strains differentially influenced the host immune response and granuloma development.

**Figure 2:**
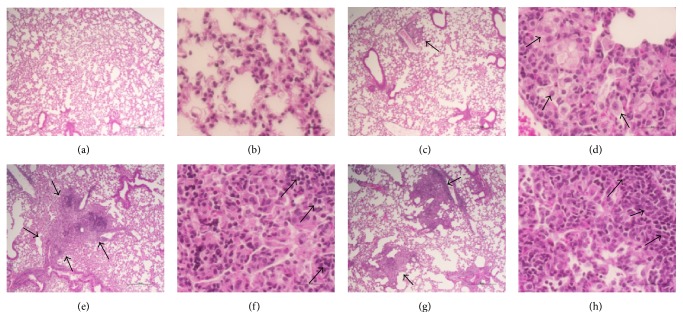
Histopathology of vaccinated and uninfected or Mtb-infected mice lungs. ((a)-(b)) H&E stained lung section of BCG vaccinated (for 8 weeks) and uninfected mice. ((c)-(d)) H&E stained lung section of Δ*bfrB* vaccinated (for 8 weeks) and uninfected mice. The arrow in (c) shows cellular aggregation. The arrows in (d) show foamy histiocytes. ((e)-(f)) H&E stained lung section of BCG vaccinated (for 8 weeks) and Mtb-infected (for 4 weeks) mice. The arrows in (e) show a multifocal, coalescent granuloma. The arrows in (f) show lymphocyte cuff at the periphery of a granuloma. ((g)-(h)) H&E stained lung section of Δ*bfrB* vaccinated (for 8 weeks) and Mtb-infected (for 4 weeks) mice. The arrows in (g) show multiple, smaller granulomas (compared to (e)). The arrows in (h) show lymphocyte cuff at the periphery of a granuloma. Magnification: 4x ((a), (c), (e), and (g)) or 40x ((b), (d), (f), and (h)).

Analysis of the lung sections at higher magnifications revealed a mostly clear and functional parenchyma without inflammation or pneumonia in the Δ*bfrB* or BCG vaccinated, uninfected animals, though very few, small, localized perivascular cellular aggregates were found in the former group (Figures 2(a)–2(d)). These cellular aggregates were comprised of foamy histiocytes and lymphocytes (Figures 2(c) and 2(d)). This indicates that a distinct lung-immune response and cell recruitment was elicited by the two vaccines. Among the Mtb-infected mice, the lungs of BCG vaccinated animals had well-organized peribronchial and perivascular granulomas that occasionally coalesce to form bigger lesions (Figure 2(e)). These highly cellular and diffused granulomas contained densely arranged foamy and nonfoamy histiocytes that appear to be macrophages and polymorphonuclear cells (PMN) at the center, surrounded by a cuff of lymphocytes at the periphery (Figure 2(f)). In contrast, the granulomatous lesions in the Δ*bfrB* vaccinated, Mtb-infected mice lungs appeared smaller, diffused, and contained more lymphocytes at the periphery of the lesions (Figures 2(g) and 2(h)). Relatively more fibrosis was also noted in these well-confined granulomas (see below). Although no necrosis was found in any of these granulomas, elevated immune cell accumulation, specifically foamy macrophages, reminiscent of lipid-pneumonia was noted in both Δ*bfrB* or BCG vaccinated, Mtb-infected mice lungs (Figures 2(e)–2(h)). Morphometric analysis of lung granulomas in the Mtb-infected mice revealed about two-fold higher lesion volume, corresponding to more lung involvement, in the BCG vaccinated, compared to the Δ*bfrB* vaccinated animals; however, the difference was not statistically significant (Supplementary Figure  3).

### 3.3. Genome-Wide Transcriptional Analysis of Δ*bfrB* or BCG Vaccinated Mtb-Infected Mice Lungs

To determine the correlates of immune response elicited in the Δ*bfrB* vaccinated Mtb-infected mice, we performed a genome-wide lung gene expression profile in these mice and compared the results with the transcriptional profile of BCG vaccinated Mtb-infected mice lungs.

**Figure 3:**
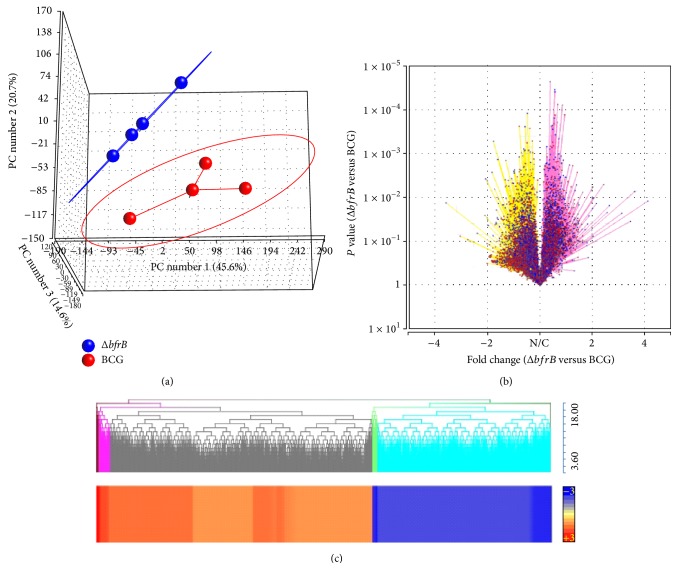
Genome-wide gene expression profiling of vaccinated and Mtb-infected mice lungs. (a) Principal component analysis of lung transcriptome data from Δ*bfrB* (blue) or BCG (red) vaccinated, Mtb-infected mice. The eclipse around each group denotes the standard deviation of the datasets. (b) Volcano plot of lung global gene expression showing the *P* value significance (*y*-axis; log scale) and fold change (*x*-axis). Upregulated genes are shaded in purple and downregulated genes are in yellow. N/C denotes no change. Each spot (blue) in the plot corresponds to a gene. (c) Intensity plot and dendrogram of significantly differentially expressed genes in the Δ*bfrB* vaccinated, compared to BCG vaccinated, Mtb-infected mice lungs. Upregulated genes are in red and downregulated genes are in blue. The color scale bar ranges from +3 (red) to −3 (blue).

The principal component analysis (PCA) mapping showed clustering of dataset from multiple samples within each group that was distinct from each other (Figure 3(a)). The reproducibility of variance in the *x*-axis, *y*-axis, and *z*-axis as shown by PC#1, 2, and 3 were 45.6%, 20.7%, and 14.7%, respectively. Of the 35,556 probes present in the mouse microarray, 21,760 were annotated. After normalization, data from the two vaccinated groups (Δ*bfrB* versus BCG) were analyzed by one-way ANOVA and compared groupwise (Figure 3(b)). Using 5% false discovery rate (FDR) (*Q* value < 0.05) as significance cut-off, we identified 1,545 significantly differentially expressed genes (SDEG). Of these SDEG, about 61% were upregulated in the Δ*bfrB* vaccinated, relative to the BCG vaccinated, Mtb-infected mouse lungs (Figures 3(b) and 3(c)). The microarray gene expression data was validated with qPCR on a randomly selected list of genes (Supplementary Table  2). The pattern and directionality of expression of selected genes was consistent between microarray and qPCR, though the absolute expression levels for some genes were different, due to inherent differences in these two methodologies.

The *z*-score based functional prediction analysis of enriched set of SDEG using IPA suggested dampening of host biological functions associated with inflammation, cellular movement, and cell death and survival, while other functions such as regulation of cell morphology, lipid metabolism, lymphoid tissue structure and development, and small molecule transport were activated in the Δ*bfrB* vaccinated, relative to the BCG vaccinated, Mtb-infected mouse lungs (Table 1).

### 3.4. Gene Networks Affected in the Δ*bfrB* or BCG Vaccinated Mtb-Infected Mice Lungs

**Figure 4:**
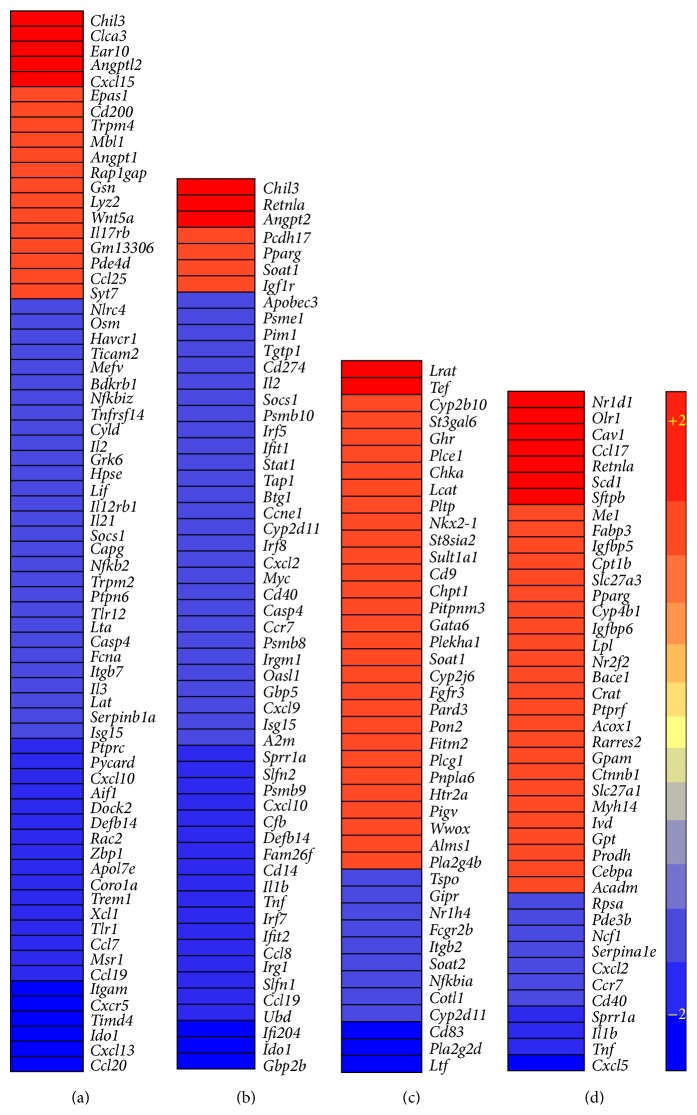
Intensity plot of network genes differentially expressed in the vaccinated and Mtb-infected mice lungs. (a) Intensity plot of significantly differentially expressed genes involved in the inflammatory response network. (b) Intensity plot of significantly differentially expressed genes involved in the STAT-1 regulon network. (c) Intensity plot of significantly differentially expressed genes involved in the PC metabolism network. (d) Intensity plot of significantly differentially expressed genes involved in the PPAR-*γ* regulon network. The values plotted in (a)–(d) are different in fold change in the Δ*bfrB* vaccinated, compared to BCG vaccinated, Mtb-infected mice lungs. Upregulated genes are in red and downregulated genes are in blue. The color scale bar ranges from +2 (red) to −2 (blue).

Among various biological functions impacted by the enriched set of SDEG, we selected the most statistically significant and potentially relevant to Tb pathogenesis for more detailed network/pathway analysis. The selected networks are inflammatory response, the STAT-1 regulon, phosphatidylcholine (PC) metabolism, and PPAR-*γ* regulon (Figure 4).

#### 3.4.1. Inflammatory Response Network

There were 70 SDEG associated with the inflammatory response that contributes to exacerbated disease pathology during Mtb infection. These genes code for cytokines, enzymes, G-protein coupled receptors, ion channels, transcriptional regulators, transmembrane receptors, and transporters. Of these, 19 were upregulated and 51 were downregulated in the Δ*bfrB *vaccinated, relative to the BCG vaccinated, Mtb-infected mouse lungs (Figure 4(a) and Supplementary Table  3). The expression pattern of SDEG in this network showed a negative *z*-score (−4.0) in the functional prediction analysis, which suggests significant downmodulation of the inflammatory response, including reduced chemotaxis and activation of leukocytes, and the acute phase response. This result is associated with and supported by our histopathology analysis that revealed smaller granulomas and reduced immunopathology in the lungs of Δ*bfrB* vaccinated, relative to the BCG vaccinated, Mtb-infected mouse lungs.

#### 3.4.2. STAT-1 Regulon Network

The signal transducer and activator of transcription-1 (STAT-1) is one of the key regulators of T-cell activation that has been shown to impact the host protective immunity during Mtb infection in humans and animal models [5–8]. We analyzed the expression pattern of SDEG that are the members of the STAT-1 regulon network, where the genes are controlling or controlled by STAT-1. Fifty-five SDEG in our dataset were involved in STAT-1 regulon network (Figure 4(b) and Supplementary Table  3). Of these, more than 87% (48 genes) were downregulated, while 7 genes were upregulated in the lungs of Δ*bfrB* vaccinated, relative to the BCG vaccinated, Mtb-infected mice. The pattern of expression of SDEG suggests inhibition of this network in the Δ*bfrB* vaccinated, Mtb-infected mice. The deactivation of STAT-1 regulon network is also associated with the downmodulation of inflammatory response in these animal lungs.

#### 3.4.3. Phosphatidylcholine Metabolism Network

Our functional prediction analysis of SDEG revealed that host lipid metabolism, specifically phosphatidylcholine (PC) biosynthesis and metabolism, was one of the top biological functions that were significantly activated (*z*-score +2.2) in the Δ*bfrB* vaccinated, relative to the BCG vaccinated, Mtb-infected mice lungs. A subset of 42 SDEG involved in the PC metabolism network were differentially regulated between Δ*bfrB* and BCG vaccinated, Mtb-infected mice lungs (Figure 4(c) and Supplementary Table  3). Of the 42 SDEG, 30 genes, including 19 (out of 20 genes) that code for enzymes, were upregulated only in the Δ*bfrB* vaccinated animals.

#### 3.4.4. PPAR-*γ* Regulon Network

One of the transcriptional regulators associated with host lipid metabolism during disease pathology is the peroxisome-proliferator-activated receptor-gamma (PPAR-*γ*) [9, 10]. Expression of* Pparγ* was upregulated in the Δ*bfrB* vaccinated, relative to the BCG vaccinated, Mtb-infected mice lungs. In addition, 15 of the PC metabolism network genes that were upregulated in the Δ*bfrB* vaccinated and Mtb-infected mice lungs were also regulated by PPAR-*γ*. Therefore, we investigated the expression pattern of PPAR-*γ* regulon network genes. These genes are either regulating or regulated by* Pparγ*. Of all the SDEG, 42 were associated with the PPAR-*γ* regulon network. Similar to the expression pattern observed for the PC metabolism network genes, more than 73% (31 genes) of the PPAR-*γ* regulon network genes were upregulated only in the Δ*bfrB* vaccinated, Mtb-infected mice lungs (Figure 4(d) and Supplementary Table  3).

Taken together, the comparative lung transcription profiling, between Δ*bfrB* and BCG vaccinated, Mtb-infected mice lungs, is strongly associated with significant downmodulation of the host inflammatory response, perhaps by deactivation of STAT-1 network and activation of host PC metabolism probably through induction of the PPAR-*γ* regulon network in the Δ*bfrB* vaccinated animals.

### 3.5. Differential Fibrosis in the Lung Granulomas of Δ*bfrB* or BCG Vaccinated Mtb-Infected Mice

Fibrosis is a cellular process important in the evolution of granulomas in Mtb-infected tissues. Resorbing and healing granulomas are reported to be encapsulated by a thick fibrotic layer, compared to an actively progressing cavitary granuloma [11]. Our histopathologic analysis of lung sections from Δ*bfrB* or BCG vaccinated, Mtb-infected mice revealed distinct cellular distribution and structural differences in the architecture of granulomas and suggested more fibrosis in the Δ*bfrB* vaccinated, compared to BCG vaccinated, Mtb-infected mice. To determine the extent of differential fibrosis between these two groups of mice, we analyzed the lung sections for collagen deposition after staining with Mason's trichrome staining method.

**Figure 5:**
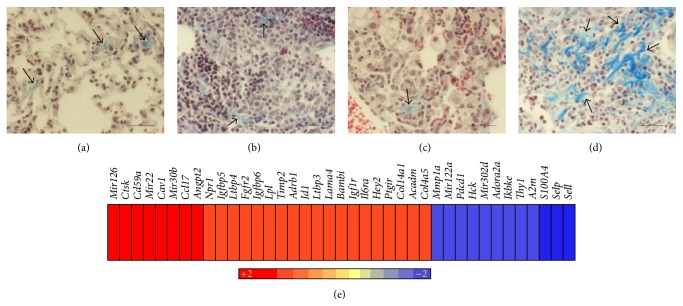
Fibrosis in the lungs of vaccinated and uninfected or Mtb-infected mice. ((a)-(b)) Masson's trichrome stained lung section of BCG vaccinated (for 8 weeks) and uninfected (a) or Mtb-infected (b) mice. ((c)-(d)) Masson's trichrome stained lung section of Δ*bfrB* vaccinated (for 8 weeks) and uninfected (c) or Mtb-infected (d) mice. The arrows in (a) and (c) show basal level of fibrosis (blue color). The arrows in (b) show minimal fibrosis in the BCG vaccinated and Mtb-infected mice. The arrows in (d) show extensive fibrosis in the Δ*bfrB* vaccinated and Mtb-infected mice. Magnification: 4x ((a) and (c)) or 40x ((b) and (d)). (e) Intensity plot of significantly differentially expressed genes involved in the fibrosis network. The values plotted are the difference in fold change in the Δ*bfrB* vaccinated, compared to BCG vaccinated, Mtb-infected mice lungs. Upregulated genes are in red and downregulated genes are in blue. The color scale bar ranges from +2 (red) to −2 (blue).

The lungs of Δ*bfrB* or BCG vaccinated, uninfected mice had similar levels of background fibrosis (Figures 5(a) and 5(c)). Among the Mtb-infected mice, those vaccinated with Δ*bfrB* had more abundant fibrosis than the BCG vaccinated animals (Figures 5(b) and 5(d)). These collagen fibers were predominantly present at the periphery of the granulomas and appeared in distinct clusters in the Δ*bfrB* vaccinated mice, compared to a more diffused pattern seen in the BCG vaccinated mice. We also interrogated the SDEG to determine the expression pattern of genes associated with fibrosis in these vaccinated and Mtb-infected mice lungs. The IPA analysis of SDEG identified a subset of 39 genes enriched for fibrosis and tissue remodeling network (Figure 5(e) and Supplementary Table  3). Of these, 27 were upregulated and 12 were downregulated in the Δ*bfrB* vaccinated, relative to the BCG vaccinated, Mtb-infected mice lungs. The expression pattern of genes predicted activation of fibrosis network in the Δ*bfrB* vaccinated Mtb-infected mice. In fact, the products of several of the upregulated genes, including* Col4a5*,* Col14a1,* and* Timp2,* are reported to be directly involved in the regulation of collagen synthesis/metabolism [12–14].

Taken together, these results suggest that the reduction in granuloma size and alleviation of lung pathology in the Δ*bfrB* vaccinated, relative to the BCG vaccinated, Mtb-infected mice lungs, is associated with increased fibrosis and wound healing processes.

### 3.6. Expression of Selected Host Immunity Related Genes in Vaccinated Animals

To gain insight on the distinctive features of the immune response induced by Δ*bfrB* and BCG vaccination, we determined the level and pattern of expression of a selected panel of 20 genes encoding various components of the immune response in the lungs of vaccinated animals (Table 2). These genes encode for proteins associated with the Th1, Th2, or Th17 type immune response, which are key components of the host response during pulmonary Mtb infection [15, 16]. The qPCR results showed similar level and directionality of expression for the majority of the tested genes, including* Il1a*,* Il1b*,* Il10*,* Il13*,* Il12a*,* Il12b*,* Il18ra*,* Tgfb*,* Timp1*,* Il1r2,* and* Nos2* in the Δ*bfrB* or BCG vaccinated mice lungs. However, expression of chemokine genes* Ccl2*,* Ccl7*,* Ccl12*,* Cxcl1*,* Cxcl3*,* Cxcl5,* and* Cxcl11* were elevated in the Δ*bfrB*, compared to BCG, vaccinated mice lungs, though the differences were not statistically significant for any of these genes (*P* > 0.05). Taken together, vaccination with Δ*bfrB* appear to elicit a similar host immune response as BCG, though elevated chemokine expression associated with cell recruitment could contribute to increased cellularity in the lungs of Δ*bfrB* vaccinated animals.

## 4. Discussion

Primary immunity to Mtb infection in mice and humans is thought to be dependent on the type-1 T helper (Th1) cell-responses that produce interferon gamma (IFN-*γ*) and tumor necrosis factor alpha (TNF*α*) which activate macrophages within the granuloma and contribute to the control of intracellular Mtb growth [17]. However, vaccine strategies that target increased Th1 response and IFN-*γ* generation did not lead to enhanced protection against Mtb infection [18]. These findings have highlighted the need to identify new correlates of protection and additional protective immune mechanisms that can be targeted to improve the efficacy of Mtb vaccines [19, 20]. In this study, we investigated the potential of the attenuated Mtb Δ*bfrB* strain to confer protection against aerosol challenge with virulent Mtb in a mouse model. Due to the effects of the* bfrB* mutation on Mtb's iron homeostasis [3], expression of genes regulated directly or indirectly by iron is altered in the Δ*bfrB* (Rodriguez G.M, unpublished). We hypothesized that the protective immune response stimulated in mice upon infection by Δ*bfrB* strain could be due to the host cell exposure to specific antigens, which might not be normally expressed by the wild type strain. Importantly, Δ*bfrB* administered subcutaneously to mice was attenuated, like BCG, supporting the safety of vaccination with this strain. The level of protection conferred by Δ*bfrB*, measured as reduction in lung bacillary load, was comparable to the standard BCG vaccination both at 4 and 8 weeks postvaccination. In addition, no significant difference was detected in the expression of a selected group of immunity related genes in the mice lungs, after vaccination with BCG or Δ*bfrB*. However, the lung histopathology and gene expression analysis, determined in animals challenged at 8 weeks postvaccination, suggested sings of an improved control of infection in Δ*bfrB* vaccinated mice, including reduced inflammation, smaller lung granulomas, and extensive fibrosis. Fibrosis is a part of tissue remodeling after injury, in which dead cells and debris accumulated during an inflammatory response are replaced. The repair process involves a regenerative phase in which injured cells are replaced by cells of the same type and a fibrosis phase in which connective tissue replaces normal tissue. In human pulmonary Tb, resorbing and healing granulomas are characterized by a fibrotic capsule that contains the infection and prevents dissemination [11]. Mature fibrotic granulomas are also associated with infection containment and resolution in some animals models of Tb [21, 22]. Though fibrosis is not commonly observed in Mtb-infected mice lungs, a differential fibrotic response was reported between resistant and susceptible strains of mice [23] and improved control of Mtb infection in the lungs of IL-10 deficient CBA/J mice was reported to be associated with fibrosis [24]. In our studies, increased fibrosis in the Δ*bfrB* vaccinated mice lungs was associated with the upregulation of fibrosis and tissue remodeling network genes, compared to BCG vaccinated animals. One of the mechanisms underlying the increased fibrosis may be linked to the upregulation of profibrotic Th2-cell responses [25] in the Δ*bfrB*, compared to BCG vaccinated, Mtb-infected mice. This is suggested by the enhanced expression of* Chil3/Ym1* encoding chitinase-3 like 1 protein, which was the most highly upregulated gene in the Δ*bfrB*-vaccinated, Mtb-infected mice and it is also upregulated by Th2 cytokines [26, 27]. In addition, Ym1 produced by dendritic cell and macrophages has Th2-inducing properties [28] and plays a critical role in inflammation, tissue remodeling, and injury. It inhibits oxidant-induced lung injury, regulates apoptosis, stimulates alternative macrophage activation, and contributes to fibrosis and wound healing. Taken together, the induction of Ym1 and its known roles in host immunity, it is possible that Ym1 and augmentation of Th2 responses may underlie the increased fibrosis and restricted disease pathology in Δ*bfrB* vaccinated, Mtb-infected mice. Future experiments will investigate that possibility.

The gene expression analyses also suggested downmodulation of inflammatory response in the Δ*bfrB* vaccinated, relative to the BCG vaccinated, Mtb-infected mice lungs. In association with this, the expression pattern of many STAT-1 mediated proinflammatory pathway genes, including* Cxcl10* (*IP-10*) and* Ccl20* that are considered biomarkers of active Tb disease, were also downregulated in the Δ*bfrB* vaccinated mice lungs [29, 30]. In contrast, expression of PC metabolism genes was upregulated in the lungs of Δ*bfrB* vaccinated mice. PC has been shown to have anti-inflammatory effects; it inhibits TNF-*α* induced proinflammatory responses, including actin filament assembly, activation of NF-*κ*B, and synthesis of proinflammatory cytokines [31].

Taken together, the modulation of disease pathology and associated gene expression profile observed in the lungs of Δ*bfrB* vaccinated, compared to BCG vaccinated Mtb-infected mice, suggests that the immune response induced by the antigenic repertoire expressed by Δ*bfrB* is distinct to that induced by BCG and it may be more effective in controlling the immunopathology during Mtb infection, thus, contributing to a better control of the disease. Further studies to determine the innate and adaptive immune response as well as cytokines and chemokines induced by Δ*bfrB* compared to BCG, will help to define specific mechanisms underlying the protection elicited by Δ*bfrB.*

## 5. Conclusions

The attenuated ferritin mutant of Mtb (Δ*bfrB*) is able to confer protection against aerosol infection with a virulent strain of Mtb. The results shown in this study suggest that, compared to BCG vaccination, reduced inflammation, limited immunopathology, and enhanced resolution of inflammation manifested as increased fibrosis are associated with the protection provided by Δ*bfrB* vaccination.

## Supplementary Material

Supplementary Figure 1. Lung bacterial load in the unvaccinated (PBS; circles) or BCG (squares) or ΔbfrB vaccinated mice at 4 (filled shapes) or 8 (open shapes) weeks.
Supplementary Figure 2. Spleen bacterial load in the unvaccinated (PBS) or BCG or ΔbfrB vaccinated mice at 4 or 8 weeks.
Supplementary Figure 3. Morphometric analysis of lung granulomas in BCG or ΔbfrB vaccinated and Mtb infected mice
Supplementary Table 1. Description of mouse gene specific oligonucleotide primers used for qPCR experiments. The accession number refers to GenBank ID.
Supplementary Table 2. Validation of microarray gene expression results by qPCR experiment with selected mouse genes. For both qPCR and microarray, the numbers show ratio of change in test gene expression, relative to Gapdh levels.
Supplementary Table 3. List of SDEG involved in inflammatory response, STAT1 regulon, PC metabolism, PPARg regulon and fibrosis network in the lungs of vaccinated and Mtb infected mice lungs.

## Figures and Tables

**Table 1:** *z*-score based functional prediction of SDEG.

Category	Functions annotation	*P* value	*z*-score	Number of molecules
*Downregulated*				
Inflammatory Response	Activation of leukocytes	3.35*E* − 12	−4	103
	Activation of myeloid cells	7.94*E* − 06	−4	39
	Activation of phagocytes	4.51*E* − 06	−3.7	45
	Activation of antigen presenting cells	2.02*E* − 06	−3.3	38
	Inflammatory response	1.24*E* − 10	−3.1	107
	Chemotaxis of granulocytes	6.58*E* − 06	−3.1	31
	Activation of mononuclear leukocytes	2.75*E* − 10	−3	77
	Chemotaxis of neutrophils	1.92*E* − 05	−3	27
	Chemotaxis of myeloid cells	5.25*E* − 07	−2.9	45
	Activation of macrophages	1.03*E* − 04	−2.9	25
	Activation of lymphocytes	2.01*E* − 10	−2.8	75
	Phagocytosis	1.91*E* − 06	−2.5	45
	Chemotaxis of leukocytes	9.02*E* − 10	−2.2	60
	Acute phase reaction	3.65*E* − 04	−2.2	7
Cellular movement	Cell movement of leukocytes	3.00*E* − 13	−2.7	122
	Lymphocyte migration	2.17*E* − 08	−2.7	55
Cell death and survival	Cell death	6.41*E* − 14	−2.6	400
*Upregulated*				
Cell morphology	Morphology of mononuclear leukocytes	1.63*E* − 10	2.9	45
	Morphology of lymphocytes	1.26*E* − 09	2.9	42
	Morphology of leukocytes	2.64*E* − 09	2.8	63
	Morphology of blood cells	1.67*E* − 07	2.8	68
	Morphology of T lymphocytes	1.85*E* − 06	2.2	26
	Morphology of B lymphocytes	3.15*E* − 05	2.1	17
Lipid metabolism	Synthesis of phosphatidylcholine	1.77*E* − 05	2.2	9
	Metabolism of phosphatidylcholine	1.16*E* − 04	2.2	10
Lymphoid tissue structure and development	Morphology of lymph follicle	2.57*E* − 10	3.4	28
	Morphology of germinal center	6.05*E* − 10	3.2	21
	Development of lymphatic system component	1.96*E* − 08	2.3	61
	Development of lymph node	2.36*E* − 08	2.9	23
	Lack of germinal center	4.84*E* − 07	3.2	11
Molecular transport	Transport of molecule	1.03*E* − 05	2.8	176

**Table 2:** Expression of selected immunity related genes in vaccinated animals.

Genes	BCG		Δ*bfrB *		Immunity type
	Mean^*^	SE	Mean^*^	SE	
*Il1a *	1.28	0.59	1.23	0.57	Th1
*Il1b *	−1.09	0.10	−1.05	0.19	Th1
*Il10 *	1.20	0.21	1.04	0.18	Th2, Th17
*Il13 *	1.11	0.14	1.16	0.15	Th2, Th17
*Il12a *	−5.86	0.10	−2.21	0.28	Th1
*Il12b *	1.18	0.38	1.17	0.51	Th1, Th17
*Il18ra *	−1.04	0.18	−1.19	0.26	Th1
*Ccl2 *	−1.09	0.11	1.13	0.07	Th17
*Ccl7 *	−8.65	0.10	1.22	0.64	Th2, Th17
*Ccl8 *	−4.12	0.17	5.34	4.82	Th2
*Ccl12 *	1.81	1.04	4.78	3.64	Th17
*Cxcl1 *	−1.72	0.38	7.13	6.25	Th17
*Cxcl3 *	−1.27	0.37	4.92	3.40	Th2
*Cxcl5 *	2.53	1.13	5.11	4.55	Th17
*Cxcl11 *	−1.03	0.12	1.01	0.08	Th1
*Tnfa *	1.13	0.59	−2.51	0.29	Th1, Th17
*Tgfb *	−1.35	0.36	−1.27	0.41	Th2
*Timp1 *	1.35	0.46	1.43	0.18	Th1, Th17
*Il1r2 *	−1.48	0.22	−1.26	0.21	Th2
*Nos2 *	−2.36	0.13	−1.03	0.39	Th1

^*∗*^Values shown are average from eight data points (duplicates of 4 animals per group).

## References

[1] Rodriguez G. M. (2006). Control of iron metabolism in *Mycobacterium tuberculosis*. *Trends in Microbiology*.

[2] Prados-Rosales R., Weinrick B. C., Piqué D. G., Jacobs W. R., Casadevall A., Rodriguez G. M. (2014). Role for *Mycobacterium tuberculosis* membrane vesicles in iron acquisition. *Journal of Bacteriology*.

[3] Pandey R., Rodriguez G. M. (2012). A ferritin mutant of Mycobacterium tuberculosis is highly susceptible to killing by antibiotics and is unable to establish a chronic infection in mice. *Infection and Immunity*.

[4] Koo M.-S., Subbian S., Kaplan G. (2012). Strain specific transcriptional response in *Mycobacterium tuberculosis* infected macrophages. *Cell Communication and Signaling*.

[5] Averbuch D., Chapgier A., Boisson-Dupuis S., Casanova J.-L., Engelhard D. (2011). The clinical spectrum of patients with deficiency of signal transducer and activator of transcription-1. *Pediatric Infectious Disease Journal*.

[6] Condos R., Raju B., Canova A. (2003). Recombinant gamma interferon stimulates signal transduction and gene expression in alveolar macrophages in vitro and in tuberculosis patients. *Infection and Immunity*.

[7] Tomlinson G. S., Cashmore T. J., Elkington P. T. G. (2011). Transcriptional profiling of innate and adaptive human immune responses to mycobacteria in the tuberculin skin test. *European Journal of Immunology*.

[8] Subbian S., Bandyopadhyay N., Tsenova L. (2013). Early innate immunity determines outcome of Mycobacterium tuberculosis pulmonary infection in rabbits. *Cell Communication and Signaling*.

[9] Kiss M., Czimmerer Z., Nagy L. (2013). The role of lipid-activated nuclear receptors in shaping macrophage and dendritic cell function: from physiology to pathology. *Journal of Allergy and Clinical Immunology*.

[10] Mahajan S., Dkhar H. K., Chandra V. (2012). *Mycobacterium tuberculosis* modulates macrophage lipid-sensing nuclear receptors PPAR*γ* and TR4 for survival. *Journal of Immunology*.

[11] Hunter R. L. (2011). Pathology of post primary tuberculosis of the lung: an illustrated critical review. *Tuberculosis*.

[12] Sand J. M., Larsen L., Hogaboam C. (2013). MMP mediated degradation of type IV collagen alpha 1 and alpha 3 chains reflects basement membrane remodeling in experimental and clinical fibrosis—validation of two novel biomarker assays. *PLoS ONE*.

[13] Fan D., Takawale A., Basu R. (2014). Differential role of TIMP2 and TIMP3 in cardiac hypertrophy, fibrosis, and diastolic dysfunction. *Cardiovascular Research*.

[14] Nakken K. E., Nygård S., Haaland T. (2007). Multiple inflammatory-, tissue remodelling- and fibrosis genes are differentially transcribed in the livers of Abcb4 (-/-) mice harbouring chronic cholangitis. *Scandinavian Journal of Gastroenterology*.

[15] Chen K., Kolls J. K. (2013). T cell-mediated host immune defenses in the lung. *Annual Review of Immunology*.

[16] Cooper A. M. (2009). Cell-mediated immune responses in tuberculosis. *Annual Review of Immunology*.

[17] Cooper A. M., Khader S. A. (2008). The role of cytokines in the initiation, expansion, and control of cellular immunity to tuberculosis. *Immunological Reviews*.

[18] Leal I. S., Smedegård B., Andersen P., Appelberg R. (2001). Failure to induce enhanced protection against tuberculosis by increasing T-cell-dependent interferon-*γ* generation. *Immunology*.

[19] Cowley S. C., Elkins K. L. (2003). CD4^+^ T cells mediate IFN-*γ*-independent control of *Mycobacterium tuberculosis* infection both in vitro and in vivo. *The Journal of Immunology*.

[20] Gallegos A. M., van Heijst J. W. J., Samstein M., Su X., Pamer E. G., Glickman M. S. (2011). A gamma interferon independent mechanism of CD4 T cell mediated control of *M. tuberculosis* infection in vivo. *PLoS Pathogens*.

[21] Gil O., Díaz I., Vilaplana C. (2010). Granuloma encapsulation is a key factor for containing tuberculosis infection in minipigs. *PLoS ONE*.

[22] Ordway D. J., Shanley C. A., Caraway M. L. (2010). Evaluation of standard chemotherapy in the guinea pig model of tuberculosis. *Antimicrobial Agents and Chemotherapy*.

[23] Marquis J.-F., Nantel A., LaCourse R., Ryan L., North R. J., Gros P. (2008). Fibrotic response as a distinguishing feature of resistance and susceptibility to pulmonary infection with *Mycobacterium tuberculosis* in mice. *Infection and Immunity*.

[24] Cyktor J. C., Carruthers B., Kominsky R. A., Beamer G. L., Stromberg P., Turner J. (2013). IL-10 inhibits mature fibrotic granuloma formation during mycobacterium tuberculosis infection. *Journal of Immunology*.

[25] Wynn T. A. (2004). Fibrotic disease and the TH1/TH2 paradigm. *Nature Reviews Immunology*.

[26] Nair M. G., Gallagher I. J., Taylor M. D. (2005). Chitinase and Fizz family members are a generalized feature of nematode infection with selective upregulation of Ym1 and Fizz1 by antigen-presenting cells. *Infection and Immunity*.

[27] Welch J. S., Escoubet-Lozach L., Sykes D. B., Liddiard K., Greaves D. R., Glass C. K. (2002). T_H_2 cytokines and allergic challenge induce Ym1 expression in macrophages by a STAT6-dependent mechanism. *The Journal of Biological Chemistry*.

[28] Arora M., Chen L., Paglia M. (2006). Simvastatin promotes Th2-type responses through the induction of the chitinase family member Ym1 in dendritic cells. *Proceedings of the National Academy of Sciences of the United States of America*.

[29] Liu M., Guo S., Hibbert J. M. (2011). CXCL10/IP-10 in infectious diseases pathogenesis and potential therapeutic implications. *Cytokine & Growth Factor Reviews*.

[30] Lee J.-S., Lee J.-Y., Son J. W. (2008). Expression and regulation of the CC-chemokine ligand 20 during human tuberculosis. *Scandinavian Journal of Immunology*.

[31] Treede I., Braun A., Sparla R. (2007). Anti-inflammatory effects of phosphatidylcholine. *The Journal of Biological Chemistry*.

