# Supplementary material for: Vaccination with an Attenuated Ferritin Mutant Protects Mice against Virulent Mycobacterium tuberculosis
Source: J Immunol Res. 2015 Aug 3;2015:385402. doi: 10.1155/2015/385402 (PMC4539171; doi:10.1155/2015/385402)
Supplement: Supplementary file 1 — Supplementary Figure 1. Lung bacterial load in the unvaccinated (PBS; circles) or BCG (squares) or ΔbfrB vaccinated mice at 4 (filled shapes) or 8 (open shapes) weeks. Supplementary Figure 2. Spleen bacterial load in the unvaccinated (PBS) or BCG or ΔbfrB vaccinated mice at 4 or 8 weeks. Supplementary Figure 3. Morphometric analysis of lung granulomas in BCG or ΔbfrB vaccinated and Mtb infected mice Supplementary Table 1. Description of mouse gene specific oligonucleotide primers used for qPCR experiments. The accession number refers to GenBank ID. Supplementary Table 2. Validation of microarray gene expression results by qPCR experiment with selected mouse genes. For both qPCR and microarray, the numbers show ratio of change in test gene expression, relative to Gapdh levels. Supplementary Table 3. List of SDEG involved in inflammatory response, STAT1 regulon, PC metabolism, PPARg regulon and fibrosis network in the lungs of vaccinated and Mtb infected mice lungs. [file 385402.f1.pdf]

Supplementary Figure 1. Lung bacterial load in mice vaccinated for 4 or 8 weeks.

Supplementary Figure 2. Spleen bacterial load in mice vaccinated for 4 or 8 weeks.

Supplementary Figure 3. Volume of lung granulomas in vaccinated and Mtb infected mice.

Supplementary Table 1. List of oligonucleotide primers used for qPCR

Supplementary Table 2. Validation of microarray gene expression by qPCR

Supplementary Table 3. List of SDEG involved in inflammatory response, STAT1 regulon, PC metabolism, PPARg regulon and fibrosis network

Supplementary Figure 1

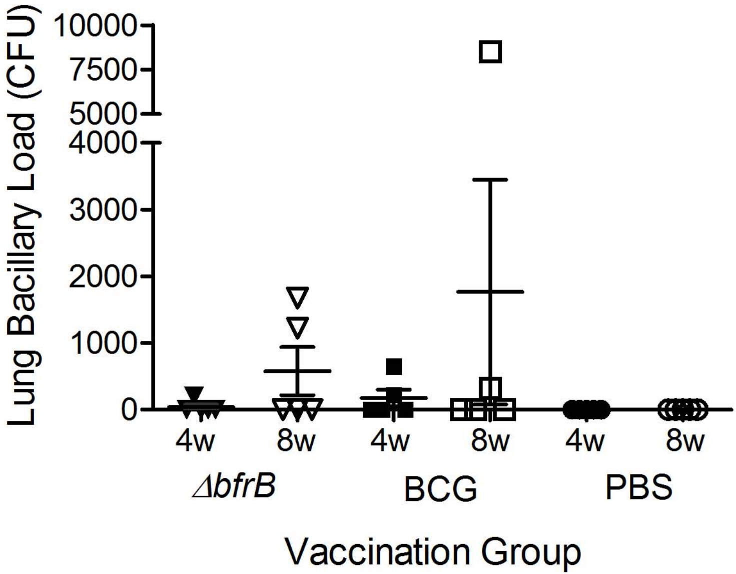

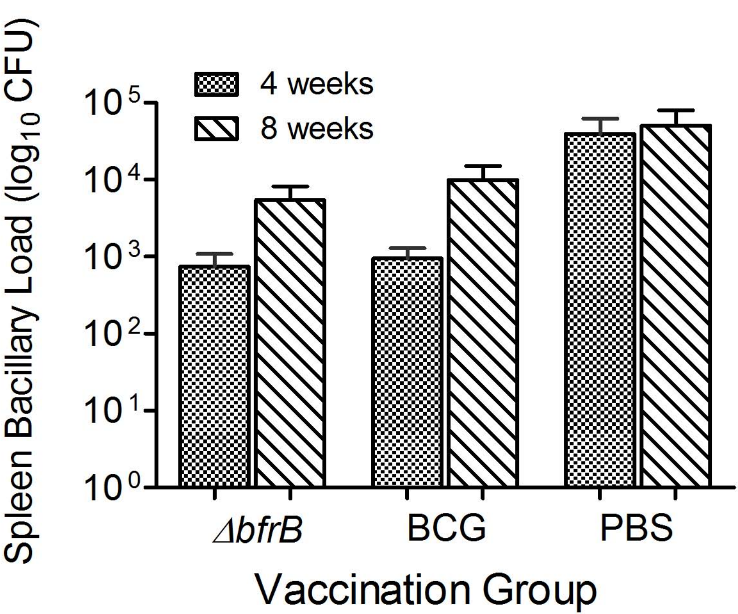

Spleen Bacillary Load (log10 CFU)
4 weeks
8 weeks
10^5
10^4
10^3
10^2
10^1
10^0
ΔbfrB
BCG
PBS
Vaccination Group

Supplementary Figure 3

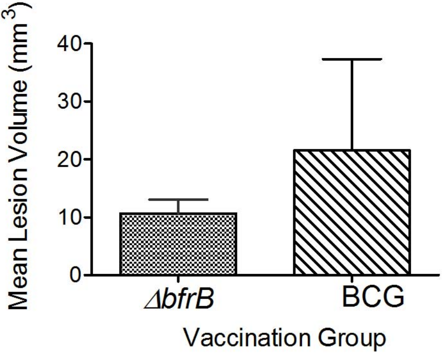

Supplementary Table 1. List of oligonucleotide primers used for qPCR

Gene	Primer pair	Sequence (5'--> 3')	Accession number
Aim1	Forward	TGCCAGAAAACAACCTTTCAGA	NM_172393
	Reverse	CTGGGTCATACCGGCTTGC	
Bank1	Forward	AGACTCAAGAGCCAAGTATCCT	NM_001033350
	Reverse	TCACCAGGTTTCTCACATGGAA	
Ccl20	Forward	GCCTCTCGTACATACAGACGC	NM_001159738
	Reverse	CCAGTTCTGCTTTGGATCAGC	
Cxcl13	Forward	GGCCACGGTATTCTGGAAGC	NM_018866
	Reverse	GGGCGTAACTTGAATCCGATCTA	
Cd14	Forward	CTCTGTCCTTAAAGCGGCTTAC	NM_009841
	Reverse	GTTGCGGAGGTTCAAGATGTT	
Cd274	Forward	GCTCCAAAGGACTTGTACGTG	NM_021893
	Reverse	TGATCTGAAGGGCAGCATTTC	
Cxcl10	Forward	CCAAGTGCTGCCGTCATTTTC	NM_021274
	Reverse	GGCTCGCAGGGATGATTTCAA	
Cxcl11	Forward	GGCTTCCTTATGTTCAAACAGGG	NM_019494
	Reverse	GCCGTTACTCGGGTAAATTAC	
Cxcl2	Forward	CCAACCACCAGGCTACAGG	NM_033166
	Reverse	GCGTCACACTCAAGCTCTG	
Cxcl9	Forward	GGAGTTCGAGGAACCCTAGTG	NM_008599
	Reverse	GGGATTTGTAGTGGATCGTGC	
Il1b	Forward	GCAACTGTTCCTGAACTCAACT	NM_008361
	Reverse	ATCTTTTGGGGTCCGTCAACT	
Il2	Forward	GTGCTCCTTGTCAACAGCG	NM_023529
	Reverse	GGGGAGTTTCAGGTTCCTGTA	
Irgm1	Forward	TGGCAATGGCATGTCATCTT	NM_008326
	Reverse	AGTACTCAGTCCGCGTCTTCGT	
Ms4a1	Forward	CCCGGCAGTGTTACTGGAG	NM_023529
	Reverse	GTGCCCATCTGGAACCAGG	
Scd1	Forward	TTCTTGCGATACACTCTGGTGC	NM_177618
	Reverse	CGGGATTGAATGTTCTTGTCGT	
Scd2	Forward	CTCTGGCGCTTACTCAGCC	NM_177292
	Reverse	AGAAGGTGGCGCTGTGATTG	
Tnfa	Forward	CTGGATGTCAATCAACAATGGGA	NM_001001495

	Reverse	ACTAGGGTGTGAGTGTTTTCTGT	
IL1a	Forward	CGAAGACTACAGTTCTGCCATT	NM_010554
	Reverse	GACGTTTCAGAGGTTCTCAGAG	
IL1b	Forward	GCAACTGTTCCTGAACTCAACT	NM_008361
	Reverse	ATCTTTTGGGGTCCGTCAACT	
IL10	Forward	GCTCTTACTGACTGGCATGAG	NM_008348
	Reverse	CGCAGCTCTAGGAGCATGTG	
IL13	Forward	CCTGGCTCTTGCTTGCCTT	NM_008356
	Reverse	GGTCTTGTGTGATGTTGCTCA	
IL12a	Forward	CTGTGCCTTGGTAGCATCTATG	NM_001159424
	Reverse	GCAGAGTCTCGCCATTATGATTC	
IL12b	Forward	TGGTTTGCCATCGTTTTGCTG	NM_008352
	Reverse	ACAGGTGAGGTTCACTGTTTCT	
IL18ra	Forward	AGACTACTTCCTGAGCACAAGA	NM_010553
	Reverse	TGTCCTTACCAATGGTTCTCACT	
Ccl2	Forward	TTAAAAACCTGGATCGGAACCAA	NM_002982.3
	Reverse	GCATTAGCTTCAGATTTACGGGT	
Ccl7	Forward	GCTGCTTTCAGCATCCAAGTG	NM_013654
	Reverse	CCAGGGACACCGACTACTG	
Ccl8	Forward	TCTACGCAGTGCTTCTTTGCC	NM_021443
	Reverse	AAGGGGGATCTTCAGCTTTAGTA	
Ccl12	Forward	ATTTCCACACTTCTATGCCTCCT	NM_011331.2
	Reverse	ATCCAGTATGGTCCTGAAGATCA	
Cxcl1	Forward	CTGGGATTCACCTCAAGAACATC	NM_008176
	Reverse	CAGGGTCAAGGCAAGCCTC	
Cxcl3	Forward	GAAGATGGTTATCGTCACCACC	NM_203320.3
	Reverse	CGTTCCAGGCATTGTACCACT	
Cxcl5	Forward	GTTCCATCTCGCCATTCATGC	NM_009141
	Reverse	GCGGCTATGACTGAGGAAGG	
Cxcl11	Forward	GGCTTCCTTATGTTCAAACAGGG	NM_019494
	Reverse	GCCGTTACTCGGGTAAATTACA	
Tgfb	Forward	CTGAGTGGCTGTCTTTTG	NM_206958
	Reverse	TTGCTGTACTGTGTGTCC	
Timp1	Forward	GCAACTCGGACCTGGTCATAA	NM_001044384
	Reverse	CGGCCCGTGATGAGAAACT	
IL1r2	Forward	GTTTCTGCTTTCACCACTCCA	NM_010555

	Reverse	GAGTCCAATTTACTCCAGGTCAG	
Nos2	Forward	GTTCTCAGCCCAACAATACAAGA	NM_194064
	Reverse	GTGGACGGGTCGATGTCAC	

Supplementary Table 2. Validation of microarray gene expression by qPCR*

S. No	Genes	qRT-PCR (mean)	sd	Microarray
1	*Aim1*	0.06	0.02	0.88
2	*Bank1*	0.06	0.03	0.50
3	*Ccl20*	0.03	0.01	0.49
4	*Cd14*	0.69	0.31	0.71
5	*Cd274*	0.20	0.13	0.85
6	*Cxcl10*	0.06	0.03	0.72
7	*Cxcl11*	0.02	0.01	0.72
8	*Cxcl13*	0.14	0.06	0.53
9	*Cxcl2*	0.03	0.01	0.79
10	*Cxcl9*	0.16	0.07	0.75
11	*Il1b*	0.10	0.01	0.70
12	*Il2*	0.01	0.01	0.85
13	*Irgml*	0.22	0.20	0.77
14	*Ms4a1*	0.06	0.02	0.48
15	*Scd1*	11.08	8.56	1.35
16	*Scd2*	1.09	0.12	1.44
17	*Tnfa*	2.9E-04	1.8E-04	0.70

*the qPCR and microarray data shown are $\log_2$ expression ratio in the Δ*bfrB* vaccinated, relative to BCG vaccinated, Mtb-infected mice.
